# Herpes Simplex Virus-2 Genital Tract Shedding Is Not Predictable over Months or Years in Infected Persons

**DOI:** 10.1371/journal.pcbi.1003922

**Published:** 2014-11-06

**Authors:** Varsha Dhankani, J. Nathan Kutz, Joshua T. Schiffer

**Affiliations:** 1 Department of Applied Mathematics, University of Washington, Seattle, Washington, United States of America; 2 Department of Medicine, University of Washington, Seattle, Washington, United States of America; 3 Vaccine and Infectious Diseases Division, Fred Hutchinson Cancer Research Center, Seattle, Washington, United States of America; 4 Clinical Research Division, Fred Hutchinson Cancer Research Center, Seattle, Washington, United States of America; Utrecht University, Netherlands

## Abstract

Herpes simplex virus-2 (HSV-2) is a chronic reactivating infection that leads to recurrent shedding episodes in the genital tract. A minority of episodes are prolonged, and associated with development of painful ulcers. However, currently, available tools poorly predict viral trajectories and timing of reactivations in infected individuals. We employed principal components analysis (PCA) and singular value decomposition (SVD) to interpret HSV-2 genital tract shedding time series data, as well as simulation output from a stochastic spatial mathematical model. Empirical and model-derived, time-series data gathered over >30 days consists of multiple complex episodes that could not be reduced to a manageable number of descriptive features with PCA and SVD. However, single HSV-2 shedding episodes, even those with prolonged duration and complex morphologies consisting of multiple erratic peaks, were consistently described using a maximum of four dominant features. Modeled and clinical episodes had equivalent distributions of dominant features, implying similar dynamics in real and simulated episodes. We applied linear discriminant analysis (LDA) to simulation output and identified that local immune cell density at the viral reactivation site had a predictive effect on episode duration, though longer term shedding suggested chaotic dynamics and could not be predicted based on spatial patterns of immune cell density. These findings suggest that HSV-2 shedding patterns within an individual are impossible to predict over weeks or months, and that even highly complex single HSV-2 episodes can only be partially predicted based on spatial distribution of immune cell density.

## Author Summary

Mathematical models are commonly used to better understand viral infections. Equations are simply rules to describe behavior of viruses, infected cells and the immune response, and are tested for their ability to reproduce serial viral levels in infected persons. Models provide insights regarding the pace of viral replication and timing of the immune response. Here we describe Herpes Simplex Virus-2 (HSV-2), an infection that defies standard modeling approaches. HSV-2 is sexually transmitted, and causes recurrent genital ulcers and frequent asymptomatic genital shedding episodes. Episodes initiate in a seemingly random fashion. Viral loads are erratic and complex during single episodes. We developed a mathematical model, which suggests that in general, shedding variability is due to heterogeneous density of immune cells in the genital tract. Yet, our model is unable to predict viral loads over time in individual patients. Here we employ several statistical tools to demonstrate that HSV-2 shedding is highly unpredictable, akin to weather patterns. Based on available spatial assessments of current viral and immunologic conditions, shedding can only be predicted over a few days, but not over ensuing weeks. These results have important clinical implications, and highlight limitations of attempting to predict outcomes in complex systems with simple mechanistic models.

## Introduction

Mechanistic mathematical models have proven to be of critical importance in identifying key features of viral infections in humans. For certain infections such as HIV, hepatitis B and hepatitis C, viral kinetics can often be recapitulated with relatively simple models that capture the balance between viral invasion, target cell depletion, and immunologic containment [1]. Results from models have driven key therapeutic insights for treatment of infection [2], [3], and overturned dogmatic principles regarding mechanisms of viral persistence [4].

Other human viral infections pose a greater challenge due to more complex replication and clearance patterns. Human herpes viruses such as cytomegalovirus (CMV), Ebstein Barr Virus (EBV) and Herpes Simplex Virus 1 and 2 (HSV-1 and 2) reactivate in an unpredictable fashion throughout the lifetime of the infected host. Of these viruses, the viral shedding patterns of HSV-2 are characterized in the greatest detail. HSV-2 is the leading cause of genital ulcers worldwide, though most infected persons are asymptomatic [5]. Shedding is sporadic but frequent [6], and consists of episodes that are enormously heterogeneous in terms of duration (1 hour to several weeks), viral titer, morphology, and symptomatic severity [7]. Individual episodes are notable for erratic and unpredictable viral trajectories. We have used mathematical models to describe the general principle that episodes initiate across regions with wide variability in density of tissue resident T-cells, and that spatial heterogeneity of immunity underlies episode variability [8]. However, the complexity and unpredictability of HSV-2 episode dynamics have precluded development of clinical tools to explain differences in shedding phenotype between infected persons [9], or to predict the subsequent course of disease in an individual over short or long time frames based on recent shedding data.

These issues are pertinent to many complex systems that exhibit behavior beyond the predictive capabilities of *a priori* mathematical models [10]. One solution to address these problems is to diminish the complexity of available data by reducing it to its most fundamental features. In fact, biologic datasets often exhibit surprisingly low-dimensional structure that can be exploited in building *a posteriori*, dimensionally reduced models that are inclusive of only the natural (dominant) variables. Such dimensionality reduction techniques, which highlight dominant dynamic features, are being developed for a very broad range of problems in the physical, engineering and biological sciences. As an example, continued success of such feature extraction techniques form the basis for modern applications in computer vision applications such as face and gesture recognition [11].

One of the most common dimensionality reduction methods is principal component analysis (PCA) which projects data onto an orthogonal set of variables or “modes” that subsequently capture the most *variance* in the data set [12]. PCA can identify a small number of independent and uncorrelated modes within complex multivariate datasets that explain variability in an existing dataset. In the case of HSV-2, this technique may identify the fundamental features underlying complex shedding patterns that are not discernable with visual inspection of time series data.

The singular value decomposition (SVD), which is at the center of the PCA reduction, allows the dimensionality of the data to be determined using least squares fitting approaches. Success of PCA relies on a relatively small number of modes that dominate and characterize the data set, such that a high percentage (generally >90%) of temporal variance in the data is captured by a relatively simple, low dimensional system. Principal components are successive modes that explain a decreasing degree of variance in the data. Once each additional mode contributes a small and equal degree of variance, additional variables are more likely to represent statistical “noise” rather than a biologic “signal” which drives fundamental dynamics of the data. With these concepts in mind, the dynamics are then projected onto a truncated set of modes with the aim of faithfully capturing and/or reproducing the dynamics observed in the entire data set. At this stage, the low-dimensional nature of the mathematical formulation can be exploited for control and/or classification of the dynamics.

Here we employ SVD to model, classify and predict the nature of HSV-2 genital tract shedding. Our results reveal that long-term shedding patterns cannot be reduced to a manageable number of variables and as such shedding phenotype cannot be predicted for an infected person over periods of weeks or months. Short-term viral behavior within a single episode can be classified with many fewer variables. We apply our analysis to simulated shedding data from a mechanistic mathematical model, and identify that the trajectory of each shedding episode appears to depend at least in part on spatial immune cell density at the time of episode initiation, though these parameters are only predictive for approximately three days. Unfortunately, density of tissue resident immune cells cannot be routinely measured in the clinic and is less predictive in our analyses for longer episodes lasting more than 3 days. To explain this lack of predictability, we demonstrate chaotic dynamics in the shedding data by computing the positive Lyapunov exponents, which measure the exponential separation of infinitesimally close trajectories of the underlying model. We therefore conclude that while mathematical models are extremely useful for explaining the biology underlying general HSV-2 shedding patterns, their use as prediction tools for infected individuals is likely to be limited.

## Results

### HSV-2 shedding patterns

**Figure 1:**
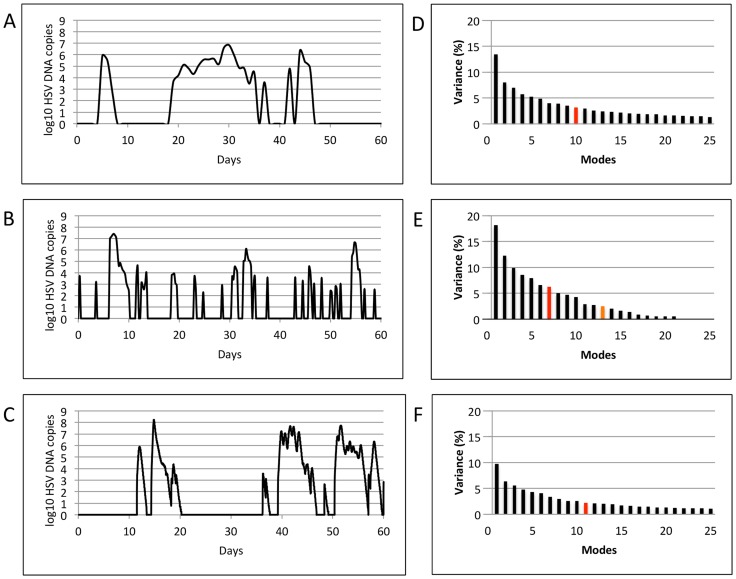
Results of Singular Value Decomposition on longitudinal data that captured multiple diverse episodes. Left column shows representative shedding curves from single patients: (a) Clinical data (swabs every 24 hours for 60 days), (b) Clinical data (swabs every 6 hours for 60 days), and (c) Modeled data (swabs every 15 minutes for 60 days). Right column (d,e,f) shows plots of singular values for the corresponding datasets (100, 25 and 98 patients, respectively). For all the three datasets, the most dominant features accounted for a very small percentage (13.5, 18.2, and 9.7 respectively) of the total variance of the system, and were closely followed by other features with gradually declining variances, making dimensional reduction infeasible. Red and orange bars indicate number of modes at which a total of 50% and 90% variance is achieved.

In order to identify fundamental dynamic patterns of HSV-2 infection, we applied Singular Value Decomposition (SVD) on shedding time series data that captured multiple diverse episodes of HSV-2 reactivation. HSV-2 infection is notable for frequent, heterogeneous spikes, or episodes, of shedding. **Figure 1** shows representative shedding curves (left column) from two datasets: (**1a**) Clinical data (100 patients, every 24 hour swabs for 60 days, [7], [13]) and (**b**) Clinical data (25 patients, every 6 hour swabs for 60 days, [14]). Each episode has a unique morphology, peak viral load and duration, but shares the characteristics of rapid viral expansion and clearance [14]). Prolonged episodes persist due to viral re-expansion following a short clearance phase.

### Mathematical model simulations

**Figure 1c** shows representative virologic output from an HSV-2 simulation model (98 simulated patients, 100 swabs/day or roughly every 15 minutes, for 60 days). The mathematical model consists of stochastic differential equations which assume that episodes are initiated randomly at various sites across the genital mucosa due to periodic release of HSV-2 from latency, and that genital tract immunity (which exhibits high spatial heterogeneity as measured by CD8+ T-cell density) wanes locally between episodes, while expanding in reference to presence of locally infected cells [8].

**Figure 2:**
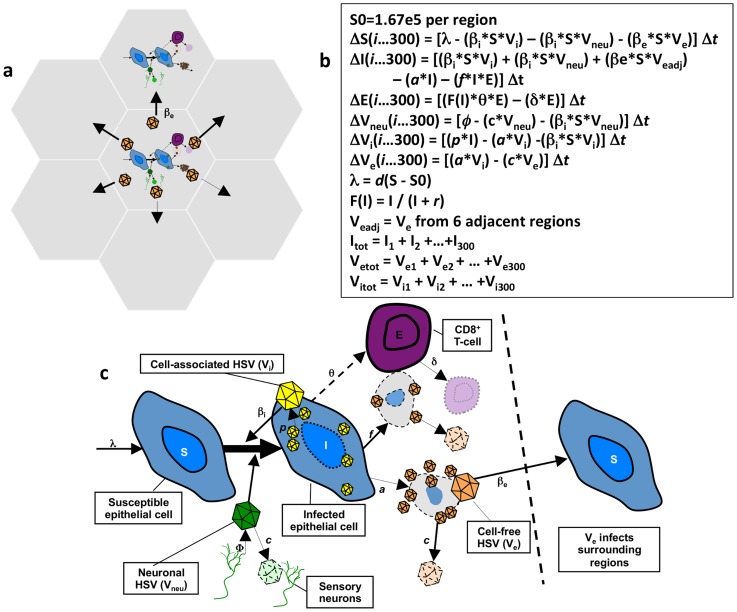
Spatial mathematical model. (a) Seven of 300 micro-regions are shown to indicate that cell-free virus from a single region can infect adjacent regions. (b) Model equations. (c) Model diagram indicating viral production, spread and local CD8+ T-cell response.

As demonstrated in **Figure 2**, the model's spatial assumptions are achieved with 300 micro-regions arranged in a hexagonal lattice. Each region is considered a battlefield in a larger war and the model allows concurrent HSV expansion and containment within multiple regions [8]. Our equations assume that HSV-2 DNA (V_neu_) is released from neurons into genital skin in a continuous steady rate (ψ* V_neu_) across wide spatial gradients: virus is randomly introduced into one of 300 regions, reflecting the wide and complex arborization pattern of neurons which release HSV-2 into genital mucosa. Episodes are initiated periodically in a single region when an epithelial cell (S) is infected with V_neu_ at rate (β_i_* V_neu_). HSV-2 replicates at rate *p* in infected cells (I). Cell-associated HSV-2 (V_i_) spreads within a single ulcer to new S with infectivity (β_i_* V_i_), leading to expansion in viral quantity. V_i_ converts to cell-free HSV (V_e_) when cells are lysed at rate *a*, or killed by CD8+ T-cells (E) at rate (f*E*I). Containment of infected cells occurs due to expansion of E at rate Θ*E within each region. Θ increases as a function of infected cells and is half maximal when I = r. E decays at rate = (δ*E) between shedding episodes. V_e_ initiates new ulcer formation in one of 6 randomly selected adjacent regions of mucosa according to rate (β_e_* V_e_). The free viral decay rate is (c* V_e_).

Parameter values are included in **Table 1**. These values were selected following testing of the model against a dataset of 1020 shedding episodes from 14,685 genital swabs performed by 531 study participants [8], [13], and allow recapitulation of population level dynamics of HSV-2 shedding including rate of episodes, early HSV expansion rate, late decay rate, heterogeneity of episode duration and peak viral production, when simulated over many years. Adjustment of parameter values leads to variability in episode and shedding rates but no change in the fundamental pattern of heterogeneous, episodic shedding [9]. Model output is consistent with clinical observations of widespread spatial spread of virus [15], [16], and localized infiltrates of HSV specific lymphocytes [17], [18], suggesting focal areas of viral replication and immunity, dispersed in multiple genital tract regions. The model's stochastic format, along with the complex nature of HSV-2 shedding data has precluded attempts to precisely reproduce or predict viral trajectories in individual infected persons.

**Table 1:** Shedding model parameter values.

βi = 6.6*10^−7^ DNA copy days/cell
βe = 1.1*10^−12^ DNA copy days/cell
*p* = 10^5^ DNA copies/cell/day
*φ* = 36 DNA copies/day
*c* = 15.8/days
Θ = 7.2/days
δ = 10^−2.6^/days
*r* = 132 infected cells at which θ is half maximal

We chose to perform SVD on model output for four reasons: first, we wished to compare dynamics in simulated and empirical datasets as a metric of mathematical model validation (if SVD provided similar results when used against empirical and simulated data); second, using model output allowed data reduction with the assumption of continuous viral sampling which is not realistic clinically; third the model allowed us to deconstruct shedding patterns over periods of times far exceeding our longest clinical protocols; finally, with the simulation model, we can exert control over key virologic and immunologic variables which are not easily measured in human experiments.

### Dimension reduction for 2-month shedding patterns

Singular value plots (**Figure 1d, e and f**) were obtained by applying SVD on corresponding datasets. Singular values quantify the percentage of variance of the system captured by a single mode (variable). The most dominant variables (corresponding to the largest singular values) accounted for only 13.5, 18.2, and 9.7 percent of the total variance of the three datasets respectively, and were closely followed by variables with gradually declining variances. To approximate these datasets to >90% accuracy required 28, 13 and 55 variables respectively, prohibiting any significant dimensional reduction. To approximate these datasets to >50% accuracy required 10, 7 and 12 variables respectively, prohibiting any significant dimensional reduction. These results imply that an individual's episode pattern over a period of two months is inherently unpredictable, and cannot be summarized with a simple set of rules. The unpredictable pattern of shedding becomes more evident with continuous sampling (more variables), probably because abrupt, frequent rebounds in viral levels within single episodes are more commonly detected with more frequent sampling, and add considerable complexity to the data (**Figure 1c**). In **Figure 1d–f**, each of the first 10 modes contributes a decreasing amount to variance, whereas beyond this point each mode contributes an equal amount to variance. These latter modes are therefore more likely to represent statistical noise.

### Dimension reduction for 10-year shedding patterns

Next, we conducted analysis of longer 10-year model simulations (98 simulated patients, 100 swabs/day or every 15 minutes) to examine whether more distinct patterns emerged from the model. We hypothesized that a longer period of sampling may lower the system's dimensionality based on the highly structured predator prey features of our simulation model [8] in which CD8+ T-cells expand as “predators” in response to surges in infected cells “prey”. The most dominant variables accounted for only 12.0 percent of the total variance of this dataset. To approximate these datasets to >90% accuracy required 14 variables, again prohibiting significant dimensional reduction, though dimensionality of 10 years of model output was notably much lower than for 60 days of model output (55 variables for >90% accuracy).

### Dimension reduction for short term shedding patterns during individual episodes

**Figure 3:**
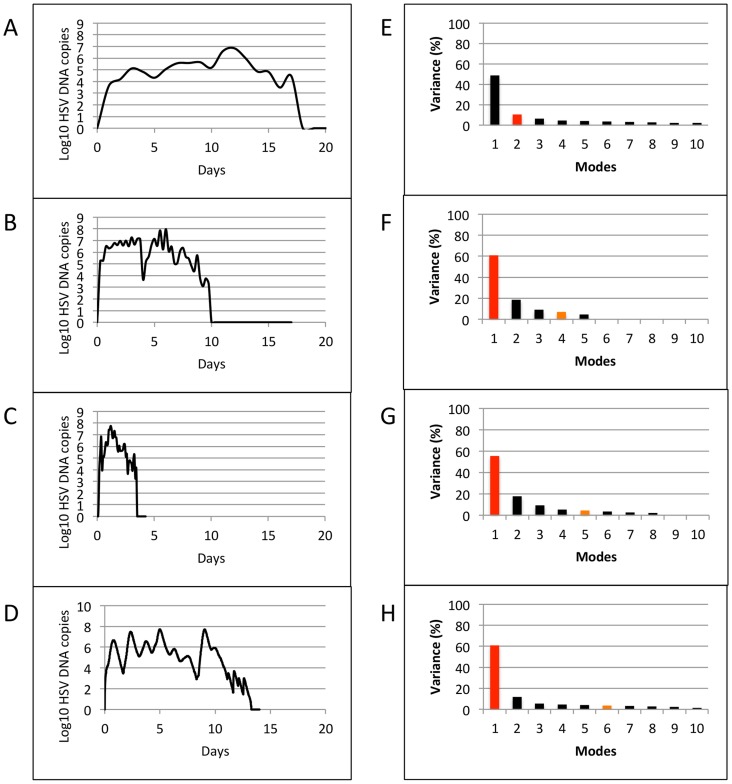
Results of Singular Value Decomposition on sets of individual non-monotonically decaying episodes. Left column shows representative curves from single patients: (a) Clinical data (23 episodes, swabs every 24 hours) (b) Clinical data (5 episodes, swabs every 6 hours), (c) Clinical data (8 episodes, 10 swabs/day: every 2 hours during the day and 4 hours overnight), and (d) Modeled data (10 episodes, 100 swabs/day). Right column (e–h) shows plots of singular values for the corresponding datasets. Red and orange bars indicate number of modes at which a total of 50% and 90% variance is achieved.

**Figure 4:**
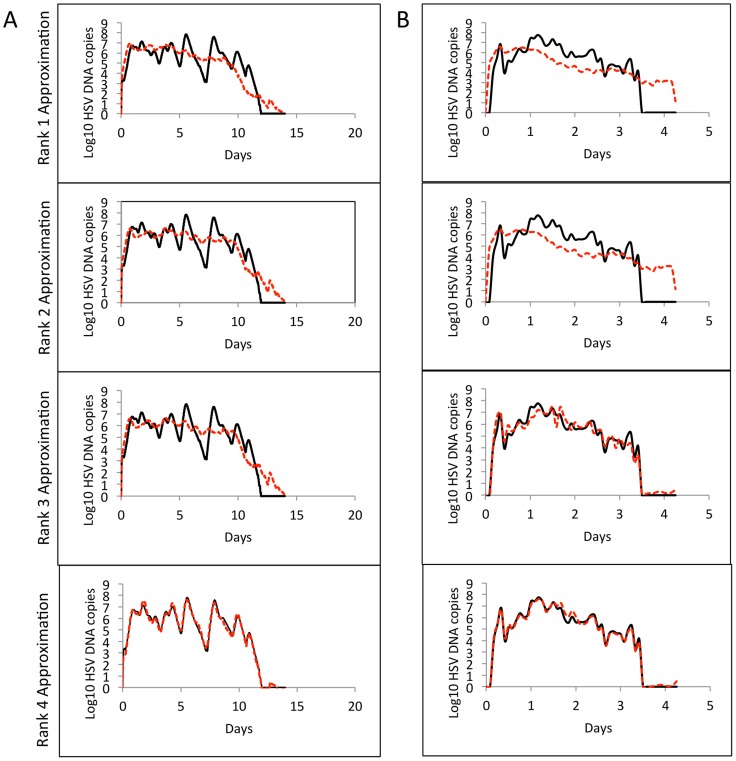
Low rank approximations for sample episodes from (a) Modeled data (10 episodes, 100 swabs/day) and (b) Clinical data (8 episodes, 10 swabs/day: every 2 hours during the day and 4 hours overnight) using the four most dominant features obtained from SVD. For the 3 clinical datasets (different sampling rates) and 1 model simulation dataset, rank-4 approximations were >85% accurate.

We next switched focus to individual episodes of viral reactivation. Many episodes last fewer than 12 hours and are characterized by single expansion and decay phases and would therefore be easily classified with a minimal number of variables. Yet, the most clinically important episodes that are typically associated with lesions are defined by multiple erratic viral re-expansions and prolonged duration (0.5–3 weeks). In order to understand viral dynamics during these non-monotonically decaying episodes, we analyzed samples from four datasets: (a) Clinical data (23 episodes, swabs every 24 hours, [7], [13]), (b) Clinical data (5 episodes, swabs every 6 hours, [14]), (c) Clinical data (8 episodes, 10 swabs/day: every 2 hours during the day and 4 hours overnight, [8]), and (d) Model simulation data (10 episodes, swabs every 15 minutes, [8]). **Figure 3** shows representative curves of non-monotonically decaying episodes from the four datasets (left column: **a, b, c and d**), and plots of singular values obtained by applying SVD on the corresponding datasets (right column: **e, f, g and h**). The results were strikingly different than that for 60-day data that captured multiple diverse episodes. When SVD was applied to sets of individual non-monotonically decaying episodes, the most dominant variables accounted for 48.6, 60.8, 55.4 and 60.9 percent of the total variance of the 4 datasets respectively. To approximate these datasets to >90% accuracy required 11, 4, 5 and 6 variables respectively. Low-rank approximations with 100% accuracy was achieved with only 6 and 9 dominant variables when applied to the every 6 hour (**Figure 3f**) as well as the 10 swabs per day episodes (**Figure 3g**). **Figure 4** demonstrates nearly perfect reproduction of single episodes generated with the mathematical model (**Fig. 4a**) and with 10 swabs per day (**Fig. 4b**) with only the 4 most dominant modes.

**Figure 5:**
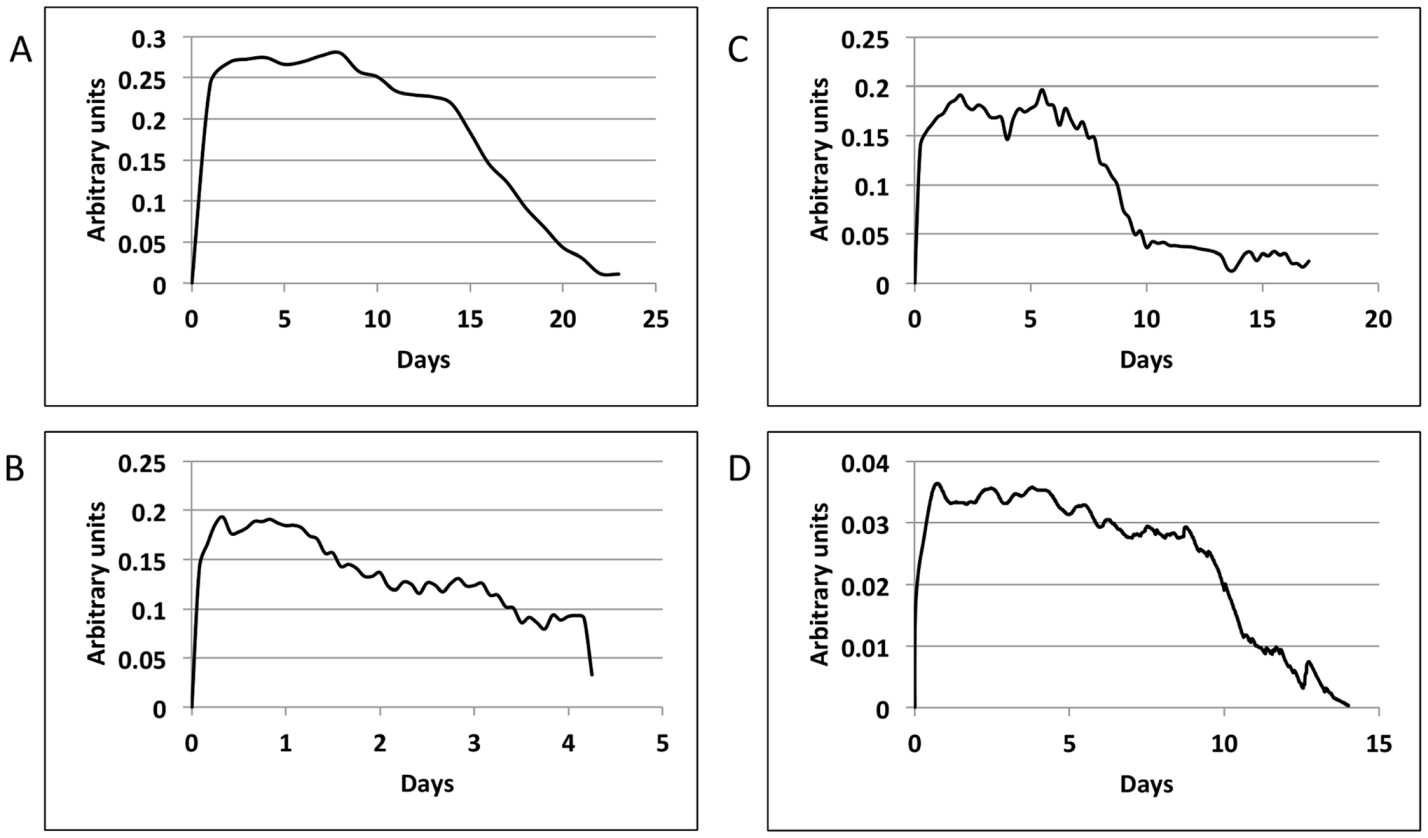
Most dominant feature of single representative non-monotonically decaying episodes. (a) Clinical data (swabs every 24 hours), (b) Clinical data (swabs every 6 hours), (c) Clinical data (10 swabs/day: every 2 hours during the day and 4 hours overnight), and (d) Modeled data (swabs every 15 minutes). The arbitrary unit is used to demonstrate relative amount of virus. All tracings demonstrate viral expansion, plateau and clearance phases.

The curves for the single most dominant variables had consistent morphologies across all the 4 datasets (**Figure 5**), indicating relatively low dimensional viral dynamics specific to non-monotonically decaying episodes. Prominent features include extremely rapid HSV-2 expansion, a variable plateau phase of viral load, and relatively slower period of viral clearance (relative to expansion). Additional modes help capture the fact that there is never actual a viral steady state, but instead a series of erratic re-expansions and peaks.

### Role of initial CD8+ T-cell densities: Linear discriminant analysis of mathematical model data

The above results imply that a set of mathematical rules may indeed predict viral trajectory of single complex episodes. Several lines of evidence suggest that immunologic conditions at the precise spatial focus of viral reactivation may determine viral trajectories within a single episode. Prior modeling demonstrated high sensitivity of episode severity to initial CD8+ T-cell density in genital mucosa [19]. These results have been confirmed in animal models in which initial conditions of infection can be rigorously controlled [20], [21]. Unfortunately, it is not possible to directly measure or control mucosal CD8+ T-cell levels in humans, as the exact site and timing of a genital reactivation is not predictable, and critical differences in immune cell density occur over spatial distances of <100 uM in tissue [8]. It is therefore necessary to capitalize on our simulation model in which initial immunologic conditions can be tracked in all spatial regions throughout the genital tract. To generate hypotheses regarding the role of the initial spatial immune densities in and around the region of episode onset in determining episode duration (and, hence, severity), we classified initial T-cell densities into 4 classes according to duration (0–2 days, 3–6 days, 7–9 days, and > = 10 days) of the ensuing episodes. We will refer to these as Classes A, B, C, and D respectively for the sake of brevity.

**Figure 6:**
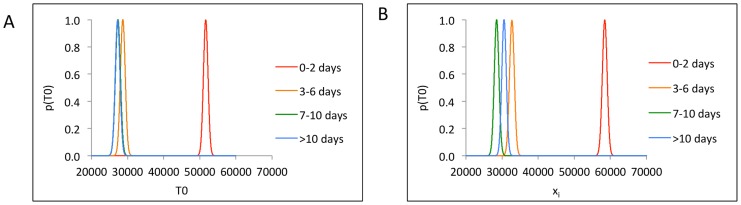
Linear Discriminant Analysis. (a) x-axis is T-cell density in the region of episode initiation (T0), (b) x-axis (x_i_) is the scalar projection value of ith initial condition <T1_i_; T2_i_; T3_i_; T4_i_; T5_i_; T6_i_> obtained by projecting onto vector w1 that maximizes Fisher criterion. In both (a) and (b), Class A (0–2 days) of initial T-cell densities showed maximum separation from the other three classes, indicating strong correlation between initial T-cell densities and episode severity only for episodes that last for 0–2 days.

In stochastic model simulations, episodes randomly initiate in one of 300 regions, each of which has a certain density of CD8+ T-cells and is surrounded by six other regions with separate densities. We attempted to correlate these regional densities with episode trajectory. Probability density curves of T-cell density in the region of episode initiation (T0) showed significant separation between Class A and the other three classes. Class A demonstrated comparatively higher T-cell densities while Classes B, C, and D showed considerable overlap in their ranges (**Figure 6a**). Class C and D were superimposed.

We next applied Linear Discriminant Analysis (LDA) on initial T-cell densities in the first ring of six model regions around the region of episode initiation (*T1*, *T2*, *T3*, *T4*, *T5*, and *T6*). A plot of probability density curves of the scalar projections obtained from LDA showed results similar to that when *T0* densities were classified. Class A projections were again well separated from Classes B, C, and D which again showed overlap (**Figure 6b**). Similar results were obtained for 4 such datasets of randomly chosen samples, indicating that surrounding region T-cell density can predict rapid HSV containment, but does not readily differentiate medium from long duration episodes. **Table 2** compares results of three different implementations of LDA and re-emphasizes the results shown in **Figure 6b**. Misclassification errors were averaged over 1000 runs with randomly chosen training and test sets. *funLDA* and MATLAB's *classify* showed comparable re-substitution and cross validation errors, whereas *dnldLDA* had better overall accuracy. For all three implementations, Class A had lower misclassification error than the other three classes. The *dnldLDA* routine achieved ∼97% accuracy in predicting Class A T-cell densities, indicating better separation of Class A T-cell densities from the other three classes.

**Table 2:** Performance of three different implementations of Linear Discriminant Analysis.

Data Set		Misclassification error (%)		
	Episode type	myLDA	dnldLDA	MATLAB's classify
**Training Set**	**Total**	46.13	29.80	44.17
	**0–2 days**	31.88	2.06	34.14
	**3–6 days**	74.80	76.57	73.08
	**7–9 days**	72.03	84.22	60.72
	**> = 10 days**	71.96	89.75	51.67
**Test Set**	**Total**	47.20	32.32	47.13
	**0–2 days**	32.46	2.72	35.85
	**3–6 days**	75.62	82.46	71.31
	**7–9 days**	75.41	90.75	65.78
	**> = 10 days**	75.09	92.16	67.35

Per class misclassification error was lower in Class A (0–2 days) than in classes B (3–6 days), C (7–9 days) and D (> = 10 days). The dnldLDA routine was >97% accurate in predicting Class A. Misclassification errors in classes B, C, and D were high for all three routines, re-emphasizing the overlap shown in **Figure 6**.

**Figure 7:**
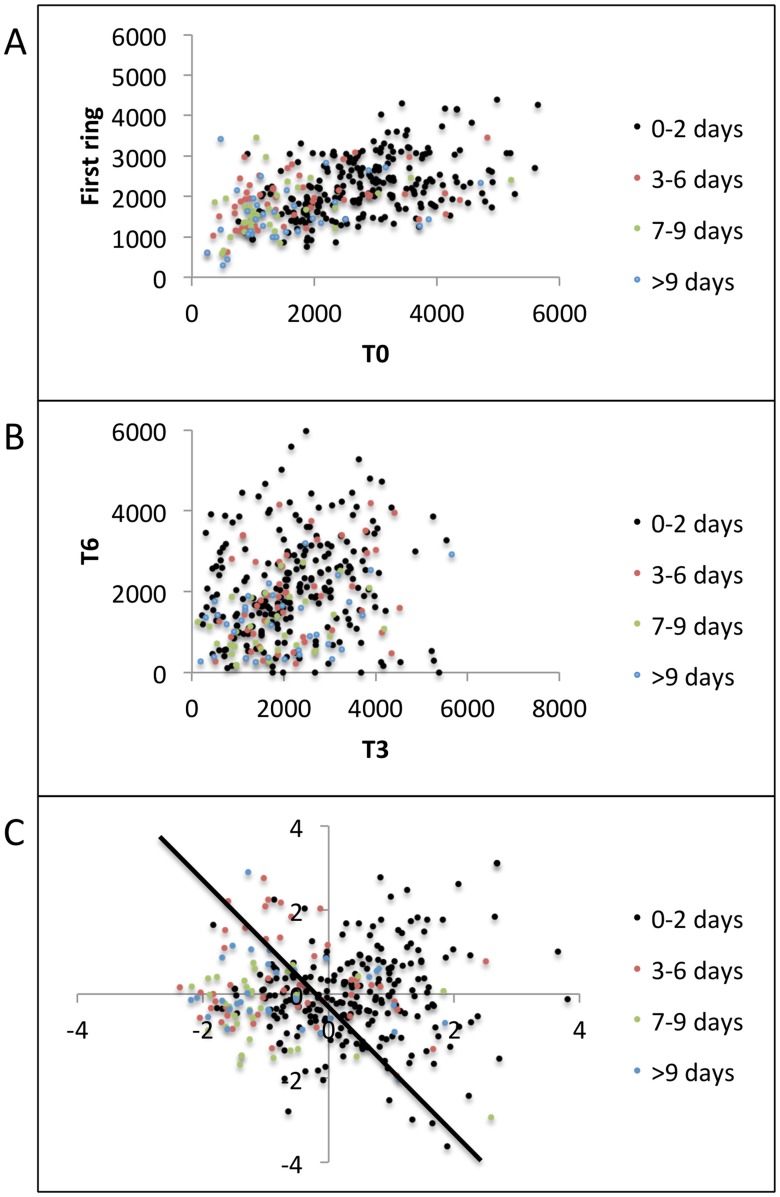
Grouped scatter plots. (a) T-cell density in region of episode initiation (T0) versus mean of the first ring of T-cell densities ((T1+T2+…+T6)/6). Class A (0–2 days) was characterized by higher T-cell densities than Classes B (3–6 days), C (7–9 days), and D (> = 10 days). Class A features were well separated in a cluster while Classes B, C, and D had overlapping feature clusters, (b) 2 randomly selected regions from the first ring reveal less separation among the 4 classes. (c) First 2 canonical features C1 and C2 obtained by projecting original features <T1; T2; T3; T4; T5; T6> onto the eigen vector given by MATLAB's manova1 routine to maximize separation between classes. Black line shows a possible decision boundary with Class A features mostly clustering on its right side, while Classes B, C, and D showing significant overlap on the left side.

Scatter plots of the features (*T0*, *T1*, *T2*, *T3*, *T4*, *T5*, and *T6*) explain LDA results. **Figure 7a** shows grouped scatter plot of *T0* vs. average first ring T-cell densities ((T1+T2+…+T6)/6) and demonstrates that higher T-cell densities in T0, and to a lesser extent in the surrounding spatial ring (T1, T2….T6), are predictive of Class A episodes more so than Classes B, C, and D. Shorter duration episodes (Class A) appeared in a cohesive cluster relative to longer duration episodes (Classes B, C, and D) which had overlapping features. We used MATLAB's *gplotmatrix* routine to generate a matrix of grouped scatter plots of pairs of these 7 features (T0, T1,…, T6). All 21 pairwise scatter plots showed similar patterns of minimal separation between episode classes (**Figure 7b**), indicating that T-cell density within single surrounding regions did not predict episode duration.

We next performed multivariate analysis of variance on the features using MATLAB's *manova1* routine. **Figure 7c** shows a grouped scatter plot of the first two canonical features C1 and C2 obtained by projecting original features onto the eigenvector that maximized separation between classes. Class A features mostly lied on the right side of the decision boundary shown in black while Classes B, C, and D had overlapping canonical features on the left side of the decision boundary.

**Figure 8:**
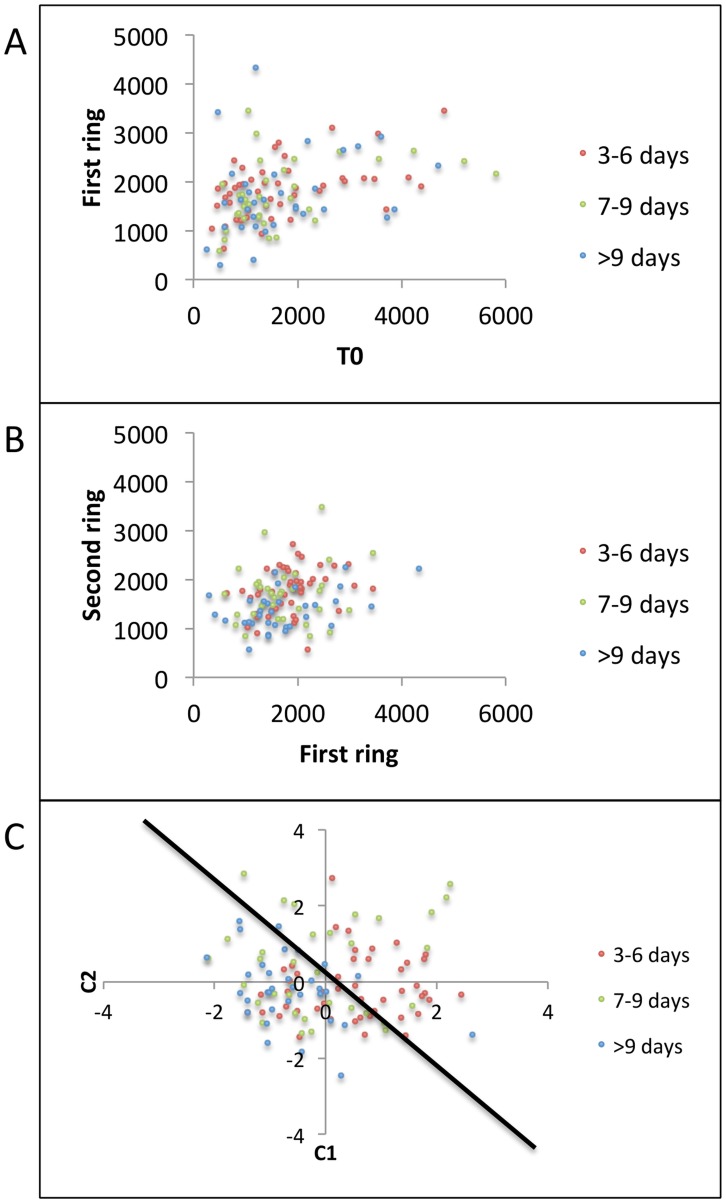
Grouped scatter plots including second ring (T7, T8,…., T18) of T-cell densities for classifying Classes B (3–6 days), C (7–9 days), and D (> = 10 days). (a) T-cell density in region of episode initiation (T0) versus mean of the first ring of T-cell densities ((T1+T2+…+T6)/6) demonstrates no separation between groups, (b) Mean of the first ring versus mean of the second ring ((T7+T8+…+T18)/12) of T-cell densities demonstrates no separation between groups. (c) First 2 canonical features C1 and C2 obtained by projecting second ring of T-cell densities onto the eigen vector given by MATLAB's manova1 routine to maximize separation between classes. Black line shows a possible decision boundary with Class D features clustering on its left side, while Classes B, and C show significant overlap on both sides.

Next, we tried to achieve separation between Classes B, C, and D by using mean of the second ring of T-cell densities ((T7+T8+…+T18)/12) around the region of episode initiation as our features for predicting severity of ensuing episodes. **Figure 8a** re-emphasizes the overlap amongst Classes B, C, and D when only *T0* and the first ring of T-cell densities were used as features. A grouped scatter plot of average first ring T-cell densities versus average second ring T-cell densities also showed similar overlap amongst Classes B, C, and D (**Figure 8b**). Grouped scatter plot of the first two canonical features obtained by multivariate analysis of variance (*MATLAB's manova1*) of the second ring of T-cell densities is shown in **Figure 8c**. Though Class D (circles) lied mostly on one side of the decision boundary, its features overlapped with Classes B and C.

Overall, these results suggest that the high T-cell density at the first site of reactivation and in surrounding regions generally predicts that viral shedding will be rapidly contained. However, the distinction between episodes of medium (3–6 days) and long (>6 days) duration cannot be determined even with simulated data in which spatial immunologic conditions are accurately identified. This result further highlights the lack of predictability in viral behavior over time frames exceeding three days.

### Naïve Bayes, support vector machines, and decision trees

Since Classes B, C, and D could not be separated with a linear decision boundary generated by LDA, we tried various advanced linear and non-linear classification techniques implemented in MATLAB. Naïve Bayes classifier is a linear classifier and assumes independence of features (http://www.mathworks.com/help/stats/naivebayes.fit.html). Using the CD8+ T-cell densities in the second ring around the reactivation site as our features, we attempted to differentiate Classes B, C, and D (medium to long duration episodes). **Table 3** shows error rates for combinations of two parameters of the naïve Bayes classifier data distribution model, and prior class probability. Errors were averaged over 1000 runs of randomly selected training and test sets (800 and 200 respectively). The lowest errors rates, obtained with kernel distribution, were ∼33% and ∼57% for training and test sets respectively, indicating poor separation between Classes B, C and D.

**Table 3:** Naïve Bayes classifier.

Prior Probabilities	Uniform		Empirical	
Distribution	Training set	Test set	Training set	Test set
**Normal**	47.47	58.00	46.78	57.28
**Kernel**	33.45	56.98	33.04	57.36

Training set and test set errors are shown for each combination of type of distribution of data and prior class probabilities. Lowest error rate is ∼33% for training set and ∼57% for test set when kernel distribution is used to model the data.

Support Vector Machines are capable of detecting non-linear decision boundaries between groups of data (http://www.mathworks.com/help/stats/support-vector-machines-svm.html). Of all the possible combinations of the parameters for support vector machines, quadratic, polynomial and Gaussian Radial basis function performed best on the training set with <1% error. However, the test set errors for all of these support vectors were ∼50%, indicating that the classifiers were over fitting the training data. **Table 4** shows average training set and test set errors for various combinations of the parameters for classification between Classes C and D. Similar misclassification errors were observed between all the three pairs of classes (B vs. C, C vs. D, and B vs. D).

**Table 4:** Support vector machines.

Kernel Function	Linear		Quadratic		Polynomial		Gaussian Radial Basis function		Multilayer Perceptron Kernel	
Method	Training	Test	Training	Test	Training	Test	Training	Test	Training	Test
**Least Squares**	35.43	54.75	0.68	44.08	0	45.85	0.21	49.08	35.58	49.61
**Quadratic Programming**	35.36	54.74	0.01	44.22	0	44.50	0.18	48.93	Fails to converge	
**Sequential Minimal Optimization**	33.38	55.87	0.88	45.31	0	45.01	1.38	49.67	62.57	56.85

Training set and test set errors are shown for each combination of kernel function and method used to find the separating hyperplane. Training set errors as low as 0% were achieved with kernel functions of higher degrees and complexities, but test set errors were always >45%, indicating model over-fitting.

Classification trees map observations (branches of the tree) about an item to its target value (leaves of the tree). Classification trees build flexible decision models and are easy to interpret. We tested a range of values for the ‘*minleaf*’ parameter for classification trees in MATLAB. For each value of the parameter, training set and test errors were averaged over 1000 randomly chosen training and test sets. The training set error increased from 0 to 28% as the minimum number of observations allowed per leaf increased from 1 to 20. Test set error, on the other hand, remained mostly constant at ∼60% for the entire range of the parameter, indicating a shift from over-fitting to under-fitting without any overall improvement in the model's predictive capacity.

### Chaos as a mechanism for unpredictable genital HSV-2 viral loads

Because the above linear and non-linear methods of dimension reduction failed to identify any significantly dominant variables in either the clinical or the model data, we sought to further understand the unpredictable nature of HSV2 trajectories. One potential explanation is chaotic dynamics, which are deterministic but sensitive to even tiny adjustments in conditions driving infection. In order to distinguish deterministic chaos from statistical noise, we computed largest Lyapunov exponents (LLE) using Rosenstein's method [22], [23]. LLEs represent the largest exponential divergence over time of initially close state-space trajectories. A positive LLE suggests chaos.

**Figure 9:**
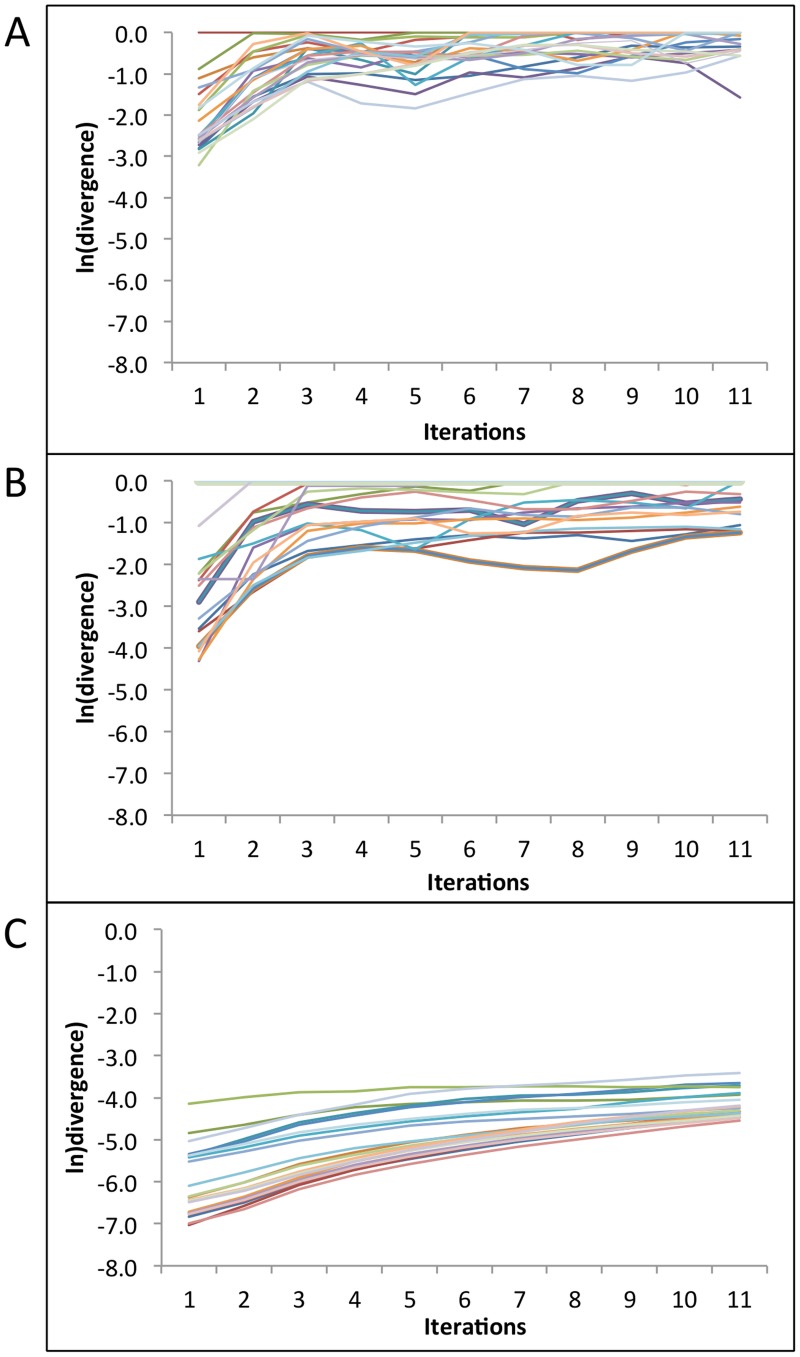
Rate of divergence of neighboring trajectories for each time series sample in (A) Clinical data (100 samples, every 24 hour swabs for 60 days), (B) Clinical data (25 samples, every 6 hour swabs for 60 days), and (C) Model simulation data (98 simulated samples, 100 swabs/day). A more prominent linear region of the curve indicates a more reliable LLE computation.

**Figure 10:**
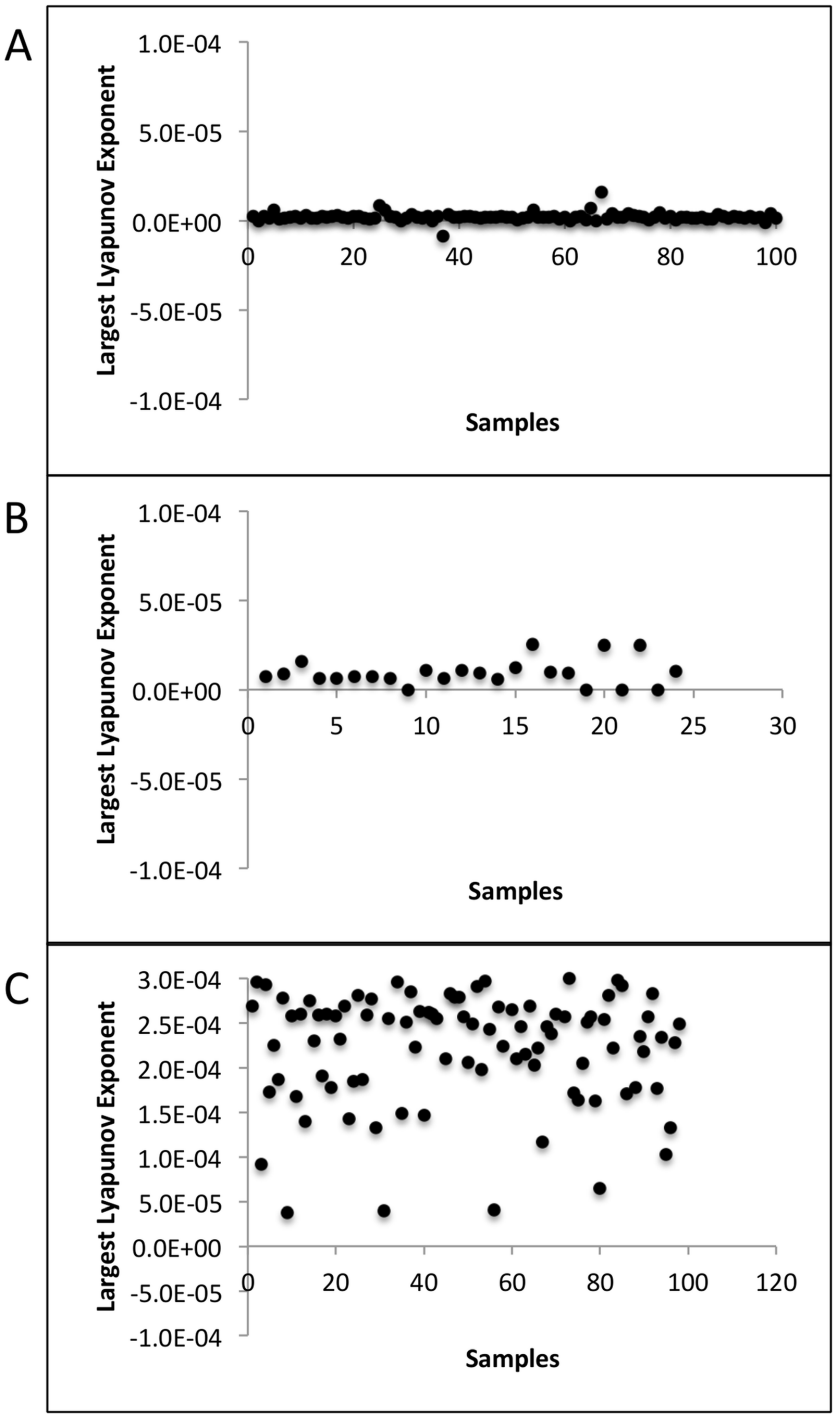
Largest Lyapunov Exponents (LLE) for each time series sample in (A) Clinical data (100 samples, every 24 hour swabs for 60 days), (B) Clinical data (25 samples, every 6 hour swabs for 60 days), and (C) Model simulation data (98 simulated samples, 100 swabs/day). LLEs were positive for all samples in data sets (B) and (C), but negative for a few samples in data set (A).

We used the TISEAN package to compute LLEs for each time series sample in two clinical sets: (a) 100 patients, every 24 hour swabs for 60 days [7] and (b) 25 patients, every 6 hour swabs for 60 days [14], as well as (c) model simulation data (98 simulated patients, 100 swabs/day [8]). **Figure 9** shows rate of change in the natural logarithm of divergence between two neighboring trajectories for each sample of the corresponding data sets. LLE is computed as the slope of the best line of fit to these curves. A more prominent linear region of the curve indicates a more reliable LLE computation [22]. **Figure 10** demonstrates that the LLEs were positive for every sample in datasets (b) and (c) but negative for a few samples with only daily sampling (a). Overall, these results suggest that viral trajectories are deterministic over timespans of months but cannot be predicted based on extreme sensitivity to immunologic and virologic conditions at onset of infection.

## Discussion

HSV-2 infection is most commonly acquired during adolescence or young adulthood and then proceeds to frequently reactivate in the human genital tract throughout the lifetime of the infected host [24]. Most infected persons are asymptomatic, but shed virus in an episodic and frequent fashion. Even in patients who do periodically develop viral ulcers, most transmissions occur during asymptomatic shedding [25]. These factors all coincide to favor an optimal transmission strategy, and help explain high global prevalence of HSV-2. Similar factors explain the success of other human herpes viruses such as EBV and CMV in sustaining high levels in human populations. In essence, these viruses and their human hosts have co-evolved over millennia to arrive at a point of compromise where shedding occurs at consistently high levels over decades, but mortality rate is extremely low. The intricate balance between HSV and infected host is further demonstrated by stable shedding rates within an individual over the course of decades [26].

Given this highly organized interplay between the virus and immune system, it is surprising that we did not identify more definitive, low dimensional patterns of viral shedding using data reduction techniques such as SVD, particularly given the fact that population level features of shedding (including episode rate, expansion and clearance slopes, duration and peak viral load) can be recapitulated with our relatively simple stochastic differential equation based model [8]. Our calculation of positive LLEs demonstrates the fact that shedding patterns in an individual are not random and follow a deterministic course. However, two factors make viral load prediction unrealistic. First, the high dimensionality of the data implies enormous biologic complexity, both in terms of the immune response and viral factors. Second, the demonstration of chaos in the data indicates high sensitivity to temporospatial starting conditions of infection that cannot be easily measured at fine enough scales for mathematical prediction. There are numerous comparable situations in which an accurate and informative set of equations can describe a complex system of interest, despite the fact that outcomes of relevance demonstrate enormous sensitivity to initial conditions and/or small shifts in system parameters. This fact underlies limitations in predicting long terms trends in weather forecasting [23], networks of interacting neurons [27], and in this case, interactions between a chronically reactivating viral pathogen and a multi-faceted, spatially dynamic human immune system.

The ramifications for HSV-2 infection are that 1) no definitive links can be made between single episodes and the timing and severity of subsequent reactivations; 2) no cyclical patterns of shedding are evident over times frames of several months; 3) current shedding is not a reliable predictor of subsequent viral activity; 4) even a detailed spatial understanding of immune cell density prior to reactivation would only provide accurate estimates of a reactivation's severity over approximately 3 days. Moreover, our results do not suggest any testable hypotheses to explain why 1–2 month shedding patterns may differ within an infected person. From the standpoint of clinical care, this is disappointing, as clinicians must continue to rely on imprecise metrics such as psychological stress as predictive tools for the next severe shedding episode [28]. Such exposure variables are hard to define, impossible to forecast, and only weakly predictive of recurrences and shedding.

If sinusoidal patterns of viral activation had been detected, then it might be implied that simple predator prey interactions between immune cells and virus exist within genital mucosa, and that drop in local immune cell intensity, predictably portends reactivation within a certain time window. Based on our repeated observations that T-cells re-accumulate in response to high levels of viral shedding, and then slowly decay when virus is absent, we believe that predator prey dynamics do play an important role in explaining shedding patterns. These dynamics may be obscured in our current analysis by the complex spatial topology of viral replication and immune response, which concurrently occurs in dozens of sites during more serious reactivations. Alternatively, time delay in CD8+ T-cell predation may be promoting chaotic dynamics that are difficult to diagnose in our current datasets. Finally, components of the immune system other than the CD8+ T-cell response are likely to impact infection, but may shift over different time and distance scales, thereby inducing enormous complexity into the system.

The lack of definitive patterns of HSV-2 shedding may also reflect the fact that balance between virus and host evolves over time scales of years rather than months. Indeed, we identified a trend of fewer variables necessary to explain the data when we applied SVD to 10 years rather than 30 days of simulated data. However, it would be necessary to apply SVD to 10 years of empirical data to confirm this trend. Moreover, fourteen variables were still necessary to describe the model simulation data, implying a reasonably high degree of unpredictability underlying HSV-2 shedding data, even over long time frames.

Characteristics that determine the outcome of single shedding episodes are captured with limited number of features. A fundamental prediction of our simulation models is that CD8+ T-cell density at the micro-anatomic reactivation site determines extent of local viral replication. This finding has been confirmed in animal models of infection [20], [21], highlighting the importance of tissue resident CD4+ and CD8+ T-cells in immediately containing infected cells. More complex features of individual episodes such as frequent viral re-expansion are also captured with only four rank approximations. Exploratory analyses based on initial conditions derived from the spatial mathematical model, suggest that T-cell density in close proximity to the site of initial HSV-2 reactivation dictates initial viral behavior, though stochastic effects become increasingly important as episodes progress. As a result, we cannot discriminate medium duration episodes (3–6 days) from longer episodes (> = 10 days) based only on initial spatial immunologic conditions. This is probably because the site and frequency at which virus seeds new regions of genital skin is randomly determined both in our model, and in reality.

Our results do not preclude identification of clinical variables associated with low and high shedding rate in different groups of patients. For instance, certain genetic signatures predict high shedding rate, as does cell-mediated immunosuppression in the form of HIV infection or organ transplantation [29], [30]. Interestingly, gender and age are less predictive of shedding rate [13]. Nevertheless, we believe that shedding trajectories will be difficult to predict over medium to long time frames within each of these subgroups.

In summary, we employ new tools to explore short, medium and long-term trends in HSV-2 genital tract shedding. While we reinforce the critical importance of local immunity on short term shedding trends, our results also highlight that attempts to develop tools, which predict viral shedding trends over longer durations must be greeted with caution, and demonstrate that not all biologic data is easily reduced to manageable parts.

## Materials and Methods

### Ethics statement

The University of Washington institutional review board approved all research. All participants provided informed consent and we conducted all research according to principles of the Declaration of Helsinki. All primary data included in the manuscript has been published elsewhere.

### Shedding model

Simulations were performed using C++ and R. The model (**Table 1**) assumes 300 micro-regions arranged in a hexagonal lattice and allows concurrent HSV expansion and containment within multiple regions [8]. Model assumptions and equations are described in the results and available in **Figure 2**.

### Data classification techniques

Code for all statistical implementations is available https://github.com/vdhankani/HSVAnalysis. We applied a range of pattern recognition and classification techniques on shedding time series data collected in the clinic as well as time series data and initial T-cell densities generated by simulation. We used MATLAB implementations for each of these methods. For Linear Discriminant Analysis (LDA), we tested and compared MATLAB's implementation, a third-party implementation (dnldLDA: http://www.mathworks.com/matlabcentral/fileexchange/29673-lda-linear-discriminant-analysis) and our own implementation (funLDA).

We applied Singular Value Decomposition (SVD) on shedding time series data that captured multiple diverse episodes of HSV-2 reactivation. SVD involves least-square fitting to the data in higher dimensions. It projects data onto an orthogonal set of variables that subsequently capture the most variance in the data set. The dynamics are then projected onto a truncated set of variables with the aim of faithfully capturing and/or reproducing the dynamics observed in the entire data set.

We applied SVD on clinical as well as modeled time series data of over 60 days with various sampling rates. When this experiment did not reveal any promising decomposition in lower dimensions, we collected model data for over 10 years to test the hypothesis that longer period of sampling may lower the system's dimensionality based on the highly structured predator prey features of our simulation model. We also applied SVD on individual complex episodes of viral reactivation to understand viral dynamics during these non-monotonically decaying episodes.

In order to gauge the role of local immunity at the reactivation site in determining the severity of ensuing episodes, we applied Linear Discriminant Analysis (LDA) on initial CD8+ T-cell densities derived from the spatial mathematical model. LDA was used to identify a linear combination of features that characterize two or more classes within a dataset. It computes an eigenvector that maximizes Fisher's quotient by maximizing between-class variance and minimizing within-class variance. Hence, the classes show maximum separation when projected onto this eigenvector. This method is suitable for dealing with continuous independent variables that are normally distributed and dependent variables that are categorical.

Multivariate analysis of variance between the 4 classes was also performed and grouped scatter plots of the first 2 canonical features were plotted to visualize the relation among the 4 classes. When the distinction between episodes of medium and long duration (Classes B, C, and D) could not be determined using the T-cell densities of only the first ring around the reactivation site, we applied the same methods to the second ring of T-cell densities hypothesizing that as the virus spreads out, local immunity in outer rings might play role in restricting viral growth.

We used LDA to look for linear decision boundaries in the data. Because LDA is limited in its power to detect non-linear patterns in complex biological systems, we tried other advanced linear and non-linear classification techniques to classify medium and long duration episodes (Classes B, C, and D) using initial CD8+ T-cell densities. We used a naive Bayes classifier as a simple probabilistic classifier based on applying Bayes' theorem with strong (naive) independence assumptions, i.e. the presence or absence of a particular feature is unrelated to the presence or absence of any other feature, given the class variable. For our case, this feature was CD8+ T-cell density in the second ring of hexagonal regions surrounding the reactivation site form the feature set. Naïve Bayes classifiers work well in practice even when the independence assumption is not valid. With MATLAB's implementation, one can specify the distribution to model the data, as well as prior probabilities of the classes in the data. We tested combinations of ranges of these two parameters as shown in **Table 3**. Errors were averaged over 1000 runs of randomly selected training and test sets with 80–20% split.

We used support Vector Machines (SVMs) in an attempt to classify data between exactly 2 classes and do so by finding the hyperplane with the largest margin that separates all data points of one class from those of the other class. The main advantage of using SVMs for our data comes from their capability of transforming data using non-linear kernels and finding hypersurfaces in the kernel space, hence allowing non-linear separation of classes. We used SVM for pairwise classification of classes B and C, C and D, and B and D. Five different kernel transformations (linear, quadratic, polynomial, Gaussian radial basis function, and multilayer perceptron kernel) and three different methods (least squares, 2-norm soft margin, sequential minimal optimization) of finding the separating hyperplane were set as parameters.

Finally, we employed classification trees, which are decision trees that map observations about an item and this item's target value. In these tree structures, leaves represent class labels and branches represent conjunctions of features that lead to those class labels. We used MATLAB's classregtree method to classify the T-cell densities in the second ring around the region of episode initiation into classes B, C, and D (http://www.mathworks.com/help/stats/classregtree.html). An important parameter of classification trees is the minimum number of observations a tree leaf must have. Too few observations per leaf might lead to over-fitting, and too many lead to under-fitting. We tested a range of values for this parameter called ‘minleaf’ in MATLAB. For each value of the parameter, re-substitution and test errors were averaged over 1000 randomly chosen training and test sets.

### Largest Lyapunov exponents to detect chaos

In order to distinguish deterministic chaos from statistical noise, we computed largest Lyapunov exponents (LLE) using Rosenstein's method [27] which is optimized to compute largest lyapunov exponents from small time series data. LLEs represent the largest exponential divergence over time of initially close state-space trajectories. A positive LLE suggests chaos. We used the implementation of Rosenstein's method provided in the TISEAN [26] package to compute LLEs for each time series sample in clinical as well as model simulation data.
